# Genomic features and its potential implication in bone oligometastatic NSCLC

**DOI:** 10.1186/s12890-023-02354-2

**Published:** 2023-02-08

**Authors:** Rongxin Liao, Guangming Yi, Lu Shen, Xiaoyue Zhang, Zaicheng Xu, Yuan Peng, Zhenzhou Yang

**Affiliations:** 1 Department of Cancer Center, Second Affiliated Hospital, Chongqing Medical University, Chongqing, 400010 China; 2 Chongqing Clinical Research Center for Geriatrics and Gerontology, Chongqing, China; 3 Geneplus-Beijing, Beijing, China

**Keywords:** NSCLC, Bone oligometastasis, Genomic profiling, Next-generation sequencing, Tumor mutational burden

## Abstract

**Objectives:**

Emerging evidence have demonstrated that oligometastatic non-small cell lung cancer (NSCLC) can achieve clinical benefit from local consolidative therapy. Bone oligometastasis is common in advanced lung cancer, but little is known about its molecular features. The purpose of our study aimed to investigate the genomic landscape bone oligometastatic NSCLC.

**Methods:**

We collected paired blood and tissue samples from 31 bone oligometastatic NSCLC patients to make a comprehensive analysis of mutations by performing next-generation sequencing.

**Results:**

A total of 186 genomic mutations were detected from 105 distinct cancer-relevant genes, with a median number of 6 alterations per tumor. The most frequently mutated genes were EGFR (58%) and TP53 (55%), followed by KRAS (16%), CDKN2A (13%) and MET (13%). The signatures related to smoking, aging, homologous recombination deficiency and APOBEC were identified as the most important mutational processes in bone oligometastasis. The median tumor mutation burden was 4.4 mutations/Mb. Altogether, genetic alterations of bone oligometastasis are highly targetable that 74.19% of patients had at least one actionable alteration that was recommended for targeted therapy based on the OncoKB evidence. Of these patients, 16.13% had two actionable alterations that could potentially benefit from a different combination of targeted drugs to achieve better outcomes.

**Conclusion:**

Our research comprehensively elucidates the genomic features of bone oligometastatic NSCLC patients, which may optimize individualized cancer treatment in the era of precision medicine.

**Supplementary Information:**

The online version contains supplementary material available at 10.1186/s12890-023-02354-2.

## Introduction

Non-small cell lung cancer (NSCLC) has the highest morbidity and mortality of all malignant tumors worldwide [1, 2]. The bone is one of the most common distant metastases in patients with advanced lung cancer [3]. Although there are a variety of treatments including bisphosphonate administration, local radiotherapy, surgery of metastases sites and systemic therapies, the median survival of lung cancer patients after bone metastases is often less than 1 year, indicating the poor prognosis [4]. With the development of lung cancer biology, an emerging area of interest to improve survival outcome is identification of oligometastatic NSCLC, which has been recognized as a unique tumor entity that may achieve clinical benefit from local consolidative therapy (LCT) [5–7]. Bone oligometastasis is defined as a clinical stage with limited number of metastases confined to the bone. This stage of the disease is characterized by an indolent state with a better prognosis, so radical multimodal therapy can be considered. The widespread application of more accurate and sensitive imaging techniques such as PET-CT and MRI has improved the detection rate of oligometastatic state in lung cancer. However, the current definition of oligometastasis is solely defined by the number of lesions, without considering the genomic background. Moreover, bone oligometastasis may have distinct molecular features due to the organ-specific nature of tumor metastasis, and its genomic profile has hardly been investigated. Therefore, it is necessary to elucidate the mutational landscape of bone-only oligometastatic NSCLC, which will provide valuable clinical and biological insights into this unique subtype of lung cancer.

Targeted therapies and immune checkpoint inhibitors (ICIs) have revolutionized the treatment landscape for advanced lung cancer. Tumor mutation burden (TMB) is a prognostic and predictive biomarker for a variety of tumors treated with immunotherapy [8]. However, little is known regarding the expression pattern of TMB in bone oligometastasis. In addition, previous studies on actionable alterations for targeted therapy have mainly focused on conventional NSCLC populations. Thus, the evidence level and frequency for actionable alterations in bone oligometastatic NSCLC also remains unknown. In the era of precision medicine, there has been an increasing emphasis on individual comprehensive treatment under the guidance of genomics in oncology. Therefore, reassessment on the landscape of actionable alterations corresponding to targeted therapies and identification of candidate predictive immunotherapy biomarkers in bone oligometastasis are important to guide the application of tyrosine kinase inhibitors (TKIs) and ICIs in these patients.

Herein, we investigated the molecular profiles of bone oligometastatic NSCLC and their correlation with TMB, as well as the distribution of actionable alterations by performing 1021-gene next-generation sequencing (NGS). Our results shall help clinicians optimize the individualized cancer treatment for bone oligometastasis, especially the application of systematic therapy such as targeted therapy and immunotherapy.

## Method and material

### Study population and sample collection

Thirty one bone oligometastatic NSCLC patients were identified in Oncology Center of the Second Affiliated Hospital of Chongqing Medical University from February 2017 to November 2020. The main criteria for selecting recruited patients were: (1) pathological confirmation of NSCLC; (2) stage IV disease according to the eighth edition of AJCC; (3) had only one metastatic lesion confined to bone for three or more months to ensure the real oligometastatic state as much as possible. The regional lymph node involvement was not counted as a metastatic site and was categorized with the primary tumor. Clinical and histopathological information were collected from electronic medical records of each patient. The ethical committee of Chongqing Medical University approved the study. Written approval was obtained from each participant prior to enrollment.

### DNA extraction and target capture sequencing

Tissue samples from each patient were all obtained from metastatic lesions and sequenced in the Geneplus-Beijing Institute (Beijing, China) using a 1021-gene-panel. All 1021 cancer-related genes were listed in the Additional file 1: Table S1. The genomic DNA in Formalin-Fixed and Paraffin-Embedded (FFPE) tumors samples was extracted by using the QIAamp DNA FFPE Tissue Kit (Qiagen, Hilden, Germany). Then, the Qubit dsDNA BR assay (Life Technologies, USA) and 1% agarose gel electrophoresis were used to detect DNA quantity and fragment distribution. 1.0 μg of tissue DNA was sheared into 300-bp fragments using a Covaris S2 ultrasonicator. Peripheral blood was collected using a Cell-Free DNA BCT Blood Collection Tube (Streck, 218,962) and centrifuged for 10 min at 2500 × g. The supernatant was transferred into a microcentrifuge tube and centrifuged for 10 min at 16,000 × g to remove the residual cell debris. Circulating free DNA (cfDNA) from plasma was separated using the MAGMAX cell-free DNA ISO Kit (life, A29319). The Qubit dsDNA HS kit (Invitrogen, Q32851) and Agilent 2100 bioanalyzer (Agilent Technologies, Santa Clara, CA, USA) were used to detect cfDNA quantity and fragment distribution. The library was constructed by KAPA DNA Library Preparation Kit. Then, the target regions from the library were enriched using a customized probe set (Integrated DNA Technologies, IDT). Finally, the Gene+Seq-2000 sequencer (Gene + technology) was used to sequence the targeted library. Quality control criteria: content of tumor cells in the tissue samples assessed under the microscope after HE staining was ≥ 10%, and the average sequencing depth was ≥ 500X. The total amount of DNA obtained from the blood sample was ≥ 15 ng, and the average sequencing depth was ≥ 4000X.

### Sequencing data analysis

Low-quality reads and end-adaptor sequences were removed by filtering the raw sequencing data. The reads were aligned to the human genome build GRCh37 using BWA (a Burrows-Wheeler aligner) [9]. Picard tools (http://broadinstitute.github.io/picard/) were used to mark PCR duplicates. Single nucleotide variations (SNVs) and small insertions and deletions were called by MuTect (version 1.1.4) [10] and GATK (version 3.4-46-gbc02625) [11], respectively. PBL sequencing results filtered germline variations. All candidate somatic alterations identified by the bioinformatics pipeline were manually reviewed using the Integrative Genomics Viewer (IGV) [12] by assessing the overall read depth per mutation site, the mapping quality of the reads, and the quality of base calls. Mutations were annotated by ANNOVAR software [13] to identify the mutant protein-coding position and filtered silent and intronic changes. The variant allele fraction (VAF) was calculated as follows: sequencing read count of altered alleles/(sequencing read count of reference alleles + sequencing read count of altered alleles) × 100%. Mutations were identified in tissue according to the following criterias: VAF ≥ 1.0%, and at least 5 high-quality reads (Mapping quality ≥ 30, phred score ≥ 30, and no paired-end reads bias).

### Mutational signature analysis

Mutation signatures were defined in each patient by analyzing both synonymous and non-synonymous somatic SNVs, including six types of base substitutions, C > A, C > G, C > T, T > A, T > C and T > G, respectively. In terms of the 3′ and 5′ flanking nucleotides of a specific mutant base, there are 96 substitution types existing in total. Potential mutational signatures were extracted in each patient using the 30 signatures recorded in the Catalogue of Somatic Mutations in Cancer (COSMIC) [14] as a reference (R package MutationalPatterns) [15]. Afterwards, we compared the relative contribution of different signatures in bone oligometastatic tumors.

### Clinical actionability: OncoKB

Precision oncology knowledge database (OncoKB) [16] was used to classify individual gene-level events according to their therapeutic implications. This clinical support tool predicts the actionability of drug based on available clinical evidence, which is continuously updated and includes emerging biomarker data for Food and Drug Administration (FDA)-approved regimens and those still under clinical trial. An evidence classification system was established to categorize potentially actionable alterations into one of four levels based on the strength of the evidence. Level 1 represents an FDA-recognized biomarker for predicting response to an FDA-approved drug. Level 2 is a standard care biomarker predictive of response to an FDA-approved drug recommended by professional guidelines. Level 3 has compelling clinical evidence to support the biomarker as being predictive of response to a drug. Level 4 has compelling biological evidence to support the biomarker as being predictive of response to a drug. Genomic alterations having therapeutic implications were defined as “actionable mutations” and corresponded to OncoKB levels of evidence 1–4. The analysis of OncoKB database was performed on September 27th, 2022.

### Statistical analysis

The somatic mutations of all patients were evaluated enrichment in the Kyoto Encyclopedia of Genes and Genomes (KEGG) and Gene ontology (GO) using ClusterProfiler package [17] to explore their biological significance. The statistical analysis was completed with SPSS 26.0 software. The Fisher's exact test or Chi-square test was performed to compare any two categorical variables, and the T-test or Mann–Whitney U-tests was used to compare any continuous variables. A two-sided *p* < 0.05 was considered statistically significant.

## Results

### Patient characteristics

Clinical characteristics of 31 bone oligometastatic NSCLC patients enrolled in this study were summarized in Table 1. The median age for all oligometastatic patients was 66 years (range, 38–81 years). Nearly half of the patients were male (16/31), and all were diagnosed with lung adenocarcinoma. Most patients were non-smokers (16/27), no family history (21/27), outside of the spine metastasis (26/31), synchronous oligometastasis (22/31) and N2–N3 lymph nodal status (26/31).

**Table 1:** Clinical characteristics of 31 bone-only oligometastatic NSCLC patients

Characteristics	No. of patients (%)
Total	31 (100.0)
*Gender*	
Male	16 (51.6)
Female	15 (48.4)
*Age (years)*	
Median (range)	66 (38–81)
*Tumor histology*	
Adenocarcinoma	31 (100)
Squamous cell carcinoma	0 (0.0)
*Smoking*	
Yes	11 (35.5)
No	16 (51.6)
Unknown	4 (12.9)
*Family history*	
Yes	6 (19.4)
No	21 (67.7)
Unknown	4 (12.9)
*Location of bone oligometastasis*	
Spine	5 (16.1)
Outside of the spine	26 (83.9)
*Type of bone oligometastasis*	
Synchronous	22 (70.9)
Metachronous	9 (29.1)
*Nodal status*	
N0-1	5 (16.1)
N2-3	26 (83.9)

### Genomic landscape of bone oligometastatic NSCLC

**Fig. 1:**
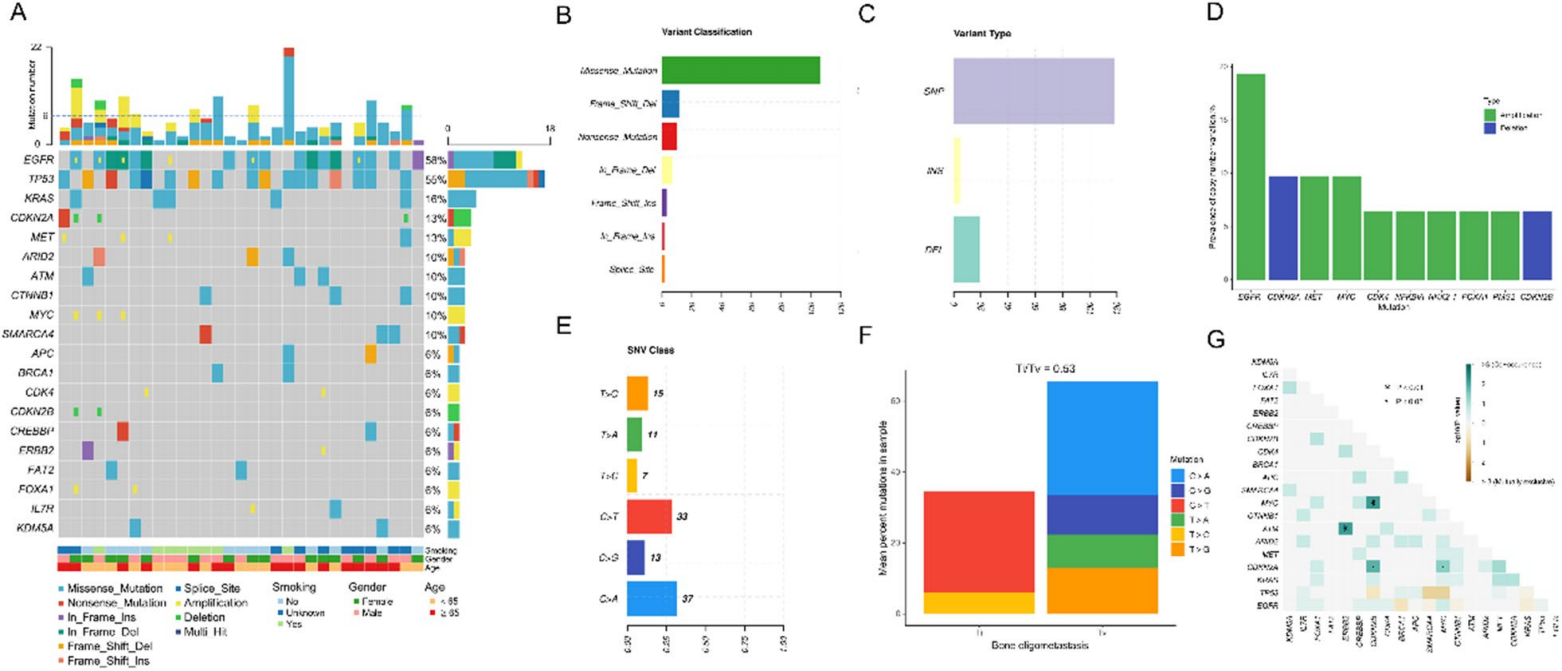
**A** Alteration landscape of 31 bone oligometastatic NSCLC patients. The heat map shows top 20 genes across all samples, with genes ranked by mutation frequency. Top bar summarizes the total number of mutations in each patient (columns), and the dashed line indicates the median number of mutations. Side bar (rows) summarizes the percentage of tumors with mutation in each gene and mutation composition for each gene in the entire cohort. Bottom heat map, smoking, gender and age information. Different colors denote different types of mutations and different clinical features. **B** Variant classification, **C** variant type, **D** prevalence of CNV alterations, **E** single nucleotide variations, **F** Ti/Tv ratios, **G** mutual exclusivity and co-occurrence analysis in bone oligometastasis

The mutational landscape of 31 bone oligometastatic NSCLC generated from targeted sequencing data is shown in Fig. 1A. The most frequently mutated genes in bone oligometastasis were EGFR (58%) and TP53 (55%). Other genomic alterations involved in decreasing order (≥ 10%), KRAS (16%), CDKN2A (13%), MET (13%), ARID2 (10%), ATM (10%), CTNNB1 (10%), MYC (10%) and SMARCA4 (10%). A total of 186 genomic mutations in 105 distinct cancer-relevant genes were identified in 31 cases. The number of alterations per sample ranged from 0 to 22, with a median of 6, and at least one mutation was found in 30 patients (96.8%) (Fig. 1A). Missense mutation was the most common mutation type, followed by frame shift deletion and nonsense (Fig. 1B). And single nucleotide polymorphisms occur more frequently than insertions and deletions (Fig. 1C). We also analyzed the prevalence of CNV changes and detected 41 clinically related CNV events in bone oligometastasis. The most frequent CNV alterations were EGFR amplification (19.35%), following by CDKN2A deletion (9.68%), MET amplification (9.68%) and MYC amplification (9.68%) (Fig. 1D). To examine the SNV spectrum, the mutational fraction of the six-base substitution for each sample was shown in Additional file 2: Fig. S1A. We found that the most frequent single nucleotide variation was C > A transversion and C > T transition, both of which are correlated with exposure to tobacco [18], and the Ti/Tv ratio of oligometastasis was 0.53 (Fig. 1E, F).

Since all the tumors of bone oligometastasis in our study were lung adenocarcinoma (LUAD), we compare the top mutated genes from our cohort with mutations in LUAD in the publicly available dataset from Memorial Sloan Kettering Cancer Center (MSKCC) (Additional file 2: Fig. S1B). The frequency of EGFR, MYC, FAT2 alterations were found to be significantly higher in oligometastasis than that reported in MSKCC. However, the significantly lower incidence of KRAS was also observed in oligometastatic group compared with MSKCC (Additional file 2: Fig. S1B). Given that EGFR and TP53 were the most common mutations in our cohort and their subtypes were closely associated with treatment decision and response to treatment, they were individually analyzed to reveal the heterogeneity. MutationMapper analysis showed that the most frequent mutation sites of EGFR and TP53 were L858R and M246L (Additional file 2: Fig. S2A and B), respectively. And the mutated sites of other high-frequency alterations (≥ 10%) were also shown in Additional file 2: Fig. S2C–I. Particularly, we observed 24 EGFR mutations in 18 patients (18/31 = 58%), with EGFR p.L858R and exon 19del accounting for 37.50% and 12.50%, respectively (Additional file 2: Fig. S1C). Moreover, TP53 was the second most frequently mutated gene in our cohort. A total of 55% (17/31) of the patients had 18 mutations in TP53, and 33% of the mutations were truncating mutations causing the inactivation of TP53. EGFR/TP53 co-alterations have been proved to reduce responsiveness to EGFR TKIs and worsen prognosis in patients with lung cancer [19–21]. Notably, the co-occurrence of EGFR and TP53 alterations comprised 61% among the EGFR-mutant oligometastatic patients (Additional file 2: Fig. S1D). Next, we further analyzed the interaction between somatic mutations in bone oligometastasis. Co-occurrence and mutual exclusivity analysis showed that ATM/ERBB2, CDKN2B/MYC, CDKN2A/CDKN2B and CDKN2A/MYC were significantly co-occurring, but we didn't detect any mutations that were mutually exclusive in oligometastatic tumors (Fig. 1G).

### Enrichment of somatic alterations by KEGG and GO analysis

**Fig. 2:**
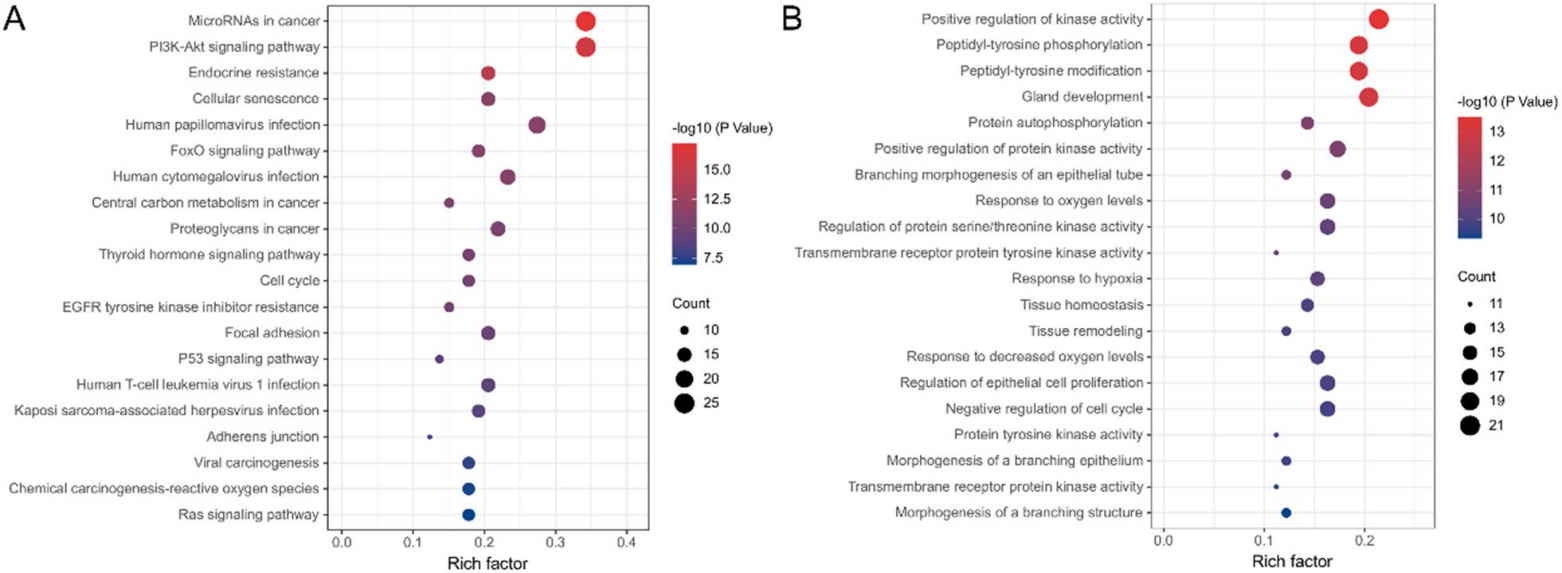
Top 20 enriched pathways by **A** KEGG and functional terms by **B** GO enrichment of somatic mutations in bone oligometastasis. Count: the number of mutations enriched in this signaling pathway or functional term

The activation or inactivation of various signaling pathways has been studied in certain types of tumors, and several cancers seem to be predisposed to activate specific signaling change that promote tumorigenesis. To elucidate the biological function of the mutations in bone oligometastasis, we performed KEGG and GO enrichment analysis. Genetic alterations including SNVs and/or copy number variations were tested for potential enrichment against each KEGG and GO pathway. Figure 2 shows the top 20 pathways enriched by KEGG (Fig. 2A) and GO (Fig. 2B) according to gene count and *p* value. Altered signaling pathways included PI3K-Akt signaling, FoxO signaling, central carbon metabolism in cancer, thyroid hormone signaling, p53 signaling, Ras signaling, and other well-known pathways. Particularly, we focused on mutations in the PI3K-Akt signaling pathway, which could promote the growth of PI3K-dependent NSCLC and enhance osteoclastogenic potential [22]. Among the patients with bone oligometastasis, 83.9% (n = 26) carried 80 genetic alterations in the PI3K-Akt pathway. We identified one truncation mutation in PTEN, leading to the loss of function of PTEN and activation of the PI3K pathway. Moreover, there were several pathways associated with viral infection, such as human papillomavirus, human cytomegalovirus, human T-cell leukemia virus 1, kaposi sarcoma-associated herpesvirus and viral carcinogenesis. Therefore, viral infection may play an important role in promoting tumorigenesis and development in bone oligometastatic NSCLC. GO enrichment analysis revealed that most of functional categories were associated with kinase activity, branching morphogenesis, response to oxygen.

### Mutational signature and TMB analysis

**Fig. 3:**
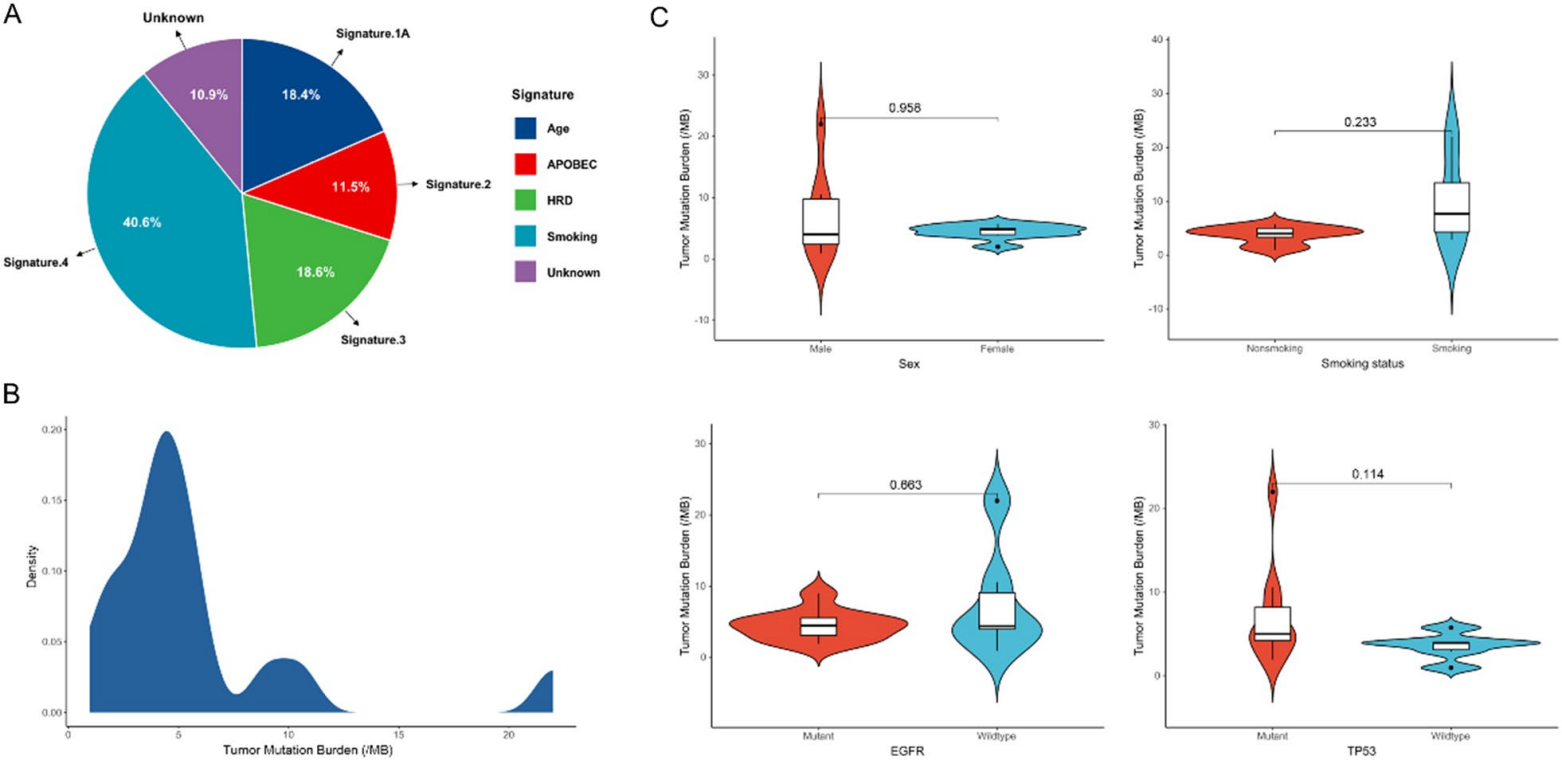
**A** Mutational signatures in bone oligometastatic NSCLC. **B** Density plot of Tumor mutational burden (TMB) in all cancer patients. **C** TMB according to gender (male versus female; smoking versus non-smoking; EGFR mutant versus EGFR wild-type; TP53 mutant versus TP53 wild-type)

The mutational signature analysis was helpful to explore the specific etiology that may contribute to the mutagenesis process of bone oligometastasis. The mutation data of all patients were classified into a base substitution matrix and analyzed using the non-negative matrix factorization method implemented in MutationalPatterns R package to infer the underlying mutational processes. We observed the mutational signature of tumor samples was composed of signature 4 (40.6%), 3 (18.6%), 1A (18.4%), 2 (11.5%) and unknown (10.9%) (Fig. 3A). Signature 4, which is associated with smoking and exposure to tobacco mutagens, was the most abundant contributor to the mutational process in oligometastasis. Signature 3 making up 18.6% of the observed signature is associated with homologous recombination deficiency (HRD). DNA damage due to HRD is also supported by the presence of somatic alterations in ATM (n = 3, 10%) and BRCA1 (n = 2, 6%) in our cohort. Signature 1A (18.4%) correlates with age and is mainly characterized by C > T transitions, which is the result of an endogenous mutational process initiated by spontaneous deamination of 5-methylcytosine. Considering the median age of our tumor samples is 66 (range, 38–81 years), which might possibly explain the abundance of signature 1A due to the older age of our patients. Signature 2 (11.5%) is linked to the activity of APOBEC cytidine deaminase. It has been proposed that the activation of these cytidine deaminases is due to viral infection, tissue inflammation or retrotransposon activity. The etiology of other mutational signature (10.9%) that contributes to bone oligometastasis still remains unknown. Taken together, apart from environmental factors such as tobacco smoking, intrinsic sources, such as HRD and APOBEC, have been described as the main cause of bone oligometastatic NSCLC.

TMB is emerging as a sensitive biomarker for immune checkpoint inhibitors, including PD-1 and PD-L1 blockade immunotherapy. In our research, the median TMB of bone oligometastasis was 4.4 mutations/Mb (range 0.96–22.00 mutations/Mb). Density plot of TMB in all cancer patients showed a long tail distribution (Fig. 3B). Of note, we identified one patient with a high TMB of 22 mutations/Mb, deviating significantly from the normal distribution. Known DNA damage repair genes, such as BRCA1 and TP53, were among the mutations in this patient. For female patients, the median TMB was 4.80 mutations/Mb, which was slightly higher than that in male patients (4.00 mutations/Mb, *p* = 0.958) (Fig. 3C). Meanwhile, the median TMB in smokers was higher than in non-smokers (7.68 vs. 4.00 mutations/Mb, *p* = 0.233) (Fig. 3C). Considering that both EGFR and TP53 mutations are top genetic variants of bone oligometastasis, we further profiled the relationship between these two alterations and TMB. The median TMB was similar between tumors with and without EGFR mutations (4.50 vs. 4.40 mutations/Mb, *p* = 0.663) (Fig. 3C). Furthermore, TP53 mutant tumors had higher median TMB than TP53 wild-type tumors (5.00 vs. 3.92 mutations/Mb, *p* = 0.114) (Fig. 3C).

### Clinically actionable genes for targeted therapy

**Fig. 4:**
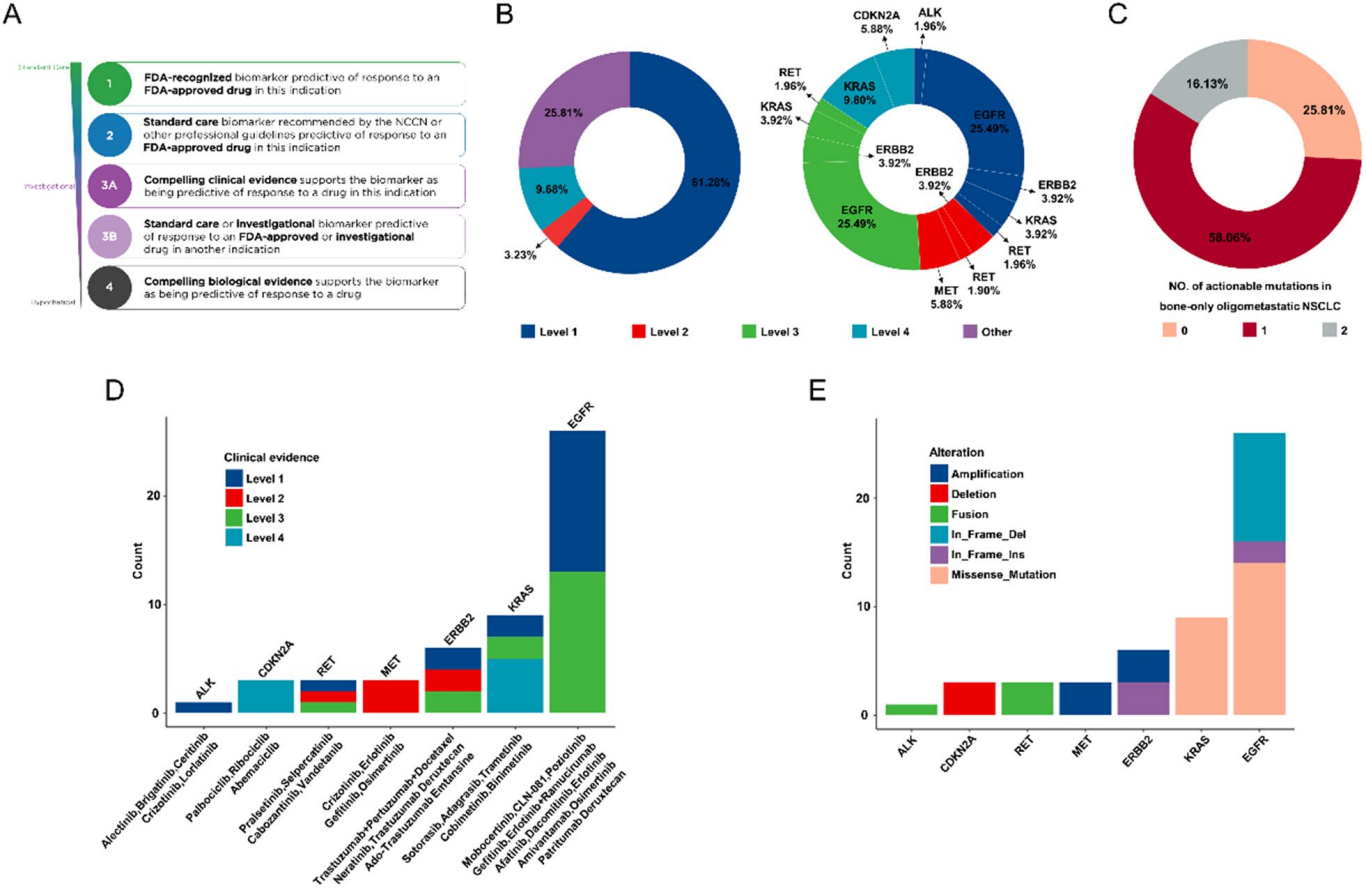
Somatic alterations identified by the 1021-panel that are clinically actionable. **A** Clinical evidence based on OncoKB was used to define alterations. **B** Samples were classified according to their highest level of actionable alterations (left). Mutated genes in different grades (right). **C** The percentage of patients with a single actionable mutation or multiple actionable mutations. **D** Distribution of levels of actionable alterations and their corresponding potential targetable drugs. **E** Distribution of alteration types of actionable genes

To assess the potential impact of genomic profiling on selecting bone oligometastasis for targeted therapy, all somatic mutations were classified into different levels based on the evidence of clinical actionability in OncoKB (Fig. 4A). Altogether, 74.19% of patients had at least one actionable alteration that was recommended for targeted therapy. Besides, 61.28% tumors had level 1 actionable mutations including ALK and RET fusion, EGFR and KRAS mutations, amplification and in-frame insertion of ERBB2. 3.23% had level 2 ERBB2, RET and MET alterations. Because the level of evidence was defined as the highest actionable targets of all mutations in each patient, no sample was assigned to level 3 since patients with level 3 actionable mutations all had higher-grade targets. Level 3 mutations include SNVs of EGFR, ERBB2 and KRAS, amplification of ERBB2 and fusion of RET. Level 4 alterations accounted for 9.68% including missense mutations of KRAS and deletions of CDKN2A (Fig. 4B, D and E). All targetable drugs corresponding to specific gene mutation in our bone oligometastatic cohort were also shown in Fig. 4E. In addition to profile of distribution of actionable mutations, we further analyzed the proportion of patients with multiple targetable gene mutations. Overall, 58.06% of patients had only one actionable alteration, while 16.13% had two actionable alterations (Fig. 4C), which may lead to better survival outcomes by using different combinations of targeted drugs.

## Discussion

Lung cancer has been recognized as a heterogeneous disease with high genomic diversity among various subtypes, leading to different treatment responses and survival outcomes [23–25]. Oligometastatic NSCLC was considered to be a distinct treatment sub-entity with unique biological and clinical features compared to conventional advanced NSCLC, including limited metastatic capacity and benefit from LCT. Bone is the most common site of distant metastasis in patients with stage IV NSCLC and has a very poor prognosis. An increasing number of clinical trials have confirmed the effectiveness and feasibility of LCT in NSCLC patients with bone oligometastasis [26, 27]. However, there is no consensus on the exact number of metastases to define oligometastasis [28]. Given that most reported studies include patients with no more than five metastases [29], we used more stringent inclusion criteria to recruit patients with only one metastatic lesion confined to bone for more than three months to ensure the real oligometastatic state. Although the treatment of oligometastasis develops rapidly, the molecular features of oligometastatic patients have not been well explored. In recent years, oncology has increasingly emphasized that therapeutic decisions of cancer should be made under the guidance of genomics. Therefore, it is necessary to conduct a comprehensive genomic analysis of such patients to provide a better insight for the precise treatment of bone oligometastasis.

In our study, we detected the most frequent genetic mutations in EGFR (58%), higher than the recent genomic studies in brain oligometastatic lung cancer (44%-47%) [30] and LUAD in Chinese (50%) [31], East Asian (47%) [32], MSKCC (33%) [33] cohorts, and the discrepancy may be due to the association of EGFR mutations with promotion of bone metastasis [34] and ethnic differences between eastern and western populations. This increase has important clinical implications because these bone oligometastatic patients are more likely to receive EGFR-TKI treatment. In particular, EGFR p.L858R and exon 19del were the predominant subtypes of EGFR mutation in our patients, but their ratio (37.5% vs 12.5%, almost 3:1) was different from another study including 2410 EGFR-mutant nonsquamous NSCLC patients (almost 1:1) [35]. Although both locations of EGFR (L858 and 19del) are canonical targets of EGFR-TKI treatment, they have been reported to respond differently to EGFR inhibitors. NSCLC patients with EGFR 19del had a better response to afatinib [36] and showed longer progression-free survival (PFS) [37] and overall survival (OS) [38] than L858R-mutant patients. Therefore, aggressive combination of LCT, such as surgery and radiotherapy, is more beneficial to improve TKI efficacy and prolong the survival of bone oligometastasis with EGFR-sensitive mutations. Moreover, TP53 was the second most common alteration in our cohort and was an independent prognostic factor in lung cancer [39]. Its co-occurrence with EGFR mutations can decrease responsiveness to EGFR TKIs and is associated with a worse prognosis [19–21]. The incidence of concurrent EGFR/TP53 in bone oligometastasis (61%) was comparable to that of EGFR-mutant NSCLC (55–65%) in other studies [20, 23, 40]. Furthermore, KRAS ranks as the third most significantly mutated gene in our samples. Previous researches have demonstrated that KRAS G12C mutations occur mostly in western patients with bone metastases, while KRAS G12V mutations occur mostly in western patients with pleural-pericardial metastases [41, 42]. Interestingly, although all samples in our cohort were lung cancer patients with metastases confined to bone, only 2 patients carried the KRAS G12C mutation but 3 patients harbored KRAS G12V mutation. This suggests that different subtypes of KRAS mutations may have a different propensity to develop metastatic sites between Chinese and western populations.

The somatic mutations of bone oligometastasis were enriched in the PI3K-Akt pathways, cell cycle, p53, and RAS pathways, which was reminiscent of the genomic mutations found in conventional NSCLC. A total of 83.9% of the patients had one or more alterations in the PI3K-Akt pathway. Previous study demonstrated that targeting the PI3K pathway could suppresses osteoclast formation in vivo and exhibit antitumour activity in mice with bone metastasis of lung cancer [22]. Considering that most bone metastases are associated with osteolysis [43], the PI3K inhibitor, buparlisib, may be a potential therapeutic strategy to prevent the structural skeletal damage correlated with bone oligometastatic NSCLC. Notably, several pathways relevant to viral infections were also enriched in our cohort, suggesting viral carcinogenesis may contribute to the development of bone oligometastasis. In particular, we focused on the human papillomavirus (HPV) infection pathway, which was reported to be connected with progression and metastasis of NSCLC. Recent studies have shown that the overexpression of HPV-16 E6 and E7 oncoproteins enhanced epithelial-mesenchymal transition (EMT) by activating STAT3 signaling pathway to promote bone metastasis in lung cancer [44]. Napabucasin, an inhibitor of intracellular STAT3, could remit bone metastases of lung cancer [45] and was approved for the treatment of gastric cancer. Many preclinical and clinical trials are validating the safety and efficacy of this drug in more advanced solid tumors [46]. Thus, napabucasin could be a treatment option for bone oligometastatic NSCLC in future. Detailing the relationship between HPV infection and bone oligometastasis may provide insight for further research into the mechanisms underlying this disease.

Mutational signatures are the cumulative result of mutational processes throughout someone's life [47]. We observed that signature 4 (smoking), signature 3 (HRD), signature 1A (age) and signature 2 (APOBEC) were identified as the most important mutational processes in samples with bone oligometastasis. Some of these signatures have shown applicability in predicting response to cancer treatment. Previously, Rizvi and colleagues found an association between signature 4 and PFS in NSCLC patients receiving Pembrolizumab [48]. Moreover, signature 2 related to APOBEC was also considered to be markedly correlated with response to ICI therapy [49]. These researches reveal the potential for qualitative analysis of the relationship between mutational patterns and treatment outcomes, but the predictive value is largely unknown in bone oligometastasis. Future studies should recruit more bone oligometastatic NSCLC patients with long-term treatment and survival follow-up data to validate the correlation between specific mutational process and efficacy response.

TMB is an emerging biomarker to predict ICI treatment response in multiple solid tumors. In our study, the median TMB of bone oligometastatic cohort was 4.4 mutations/Mb, lower than the 8.7 mutations/Mb previously reported in brain oligometastatic NSCLC [30]. This discrepancy is consistent with previous study in which TMB was higher in brain metastases compared with nonbrain metastases in NSCLC, since TMB is a site-specific biomarker with spatial distinctions [50]. In addition, TMB is widely known to be significantly associated with smoking [51], EGFR [52] and TP53 [53] in lung cancer, but we did not find any correlation between them. This may be owing to the small sample size of our cohort. Future studies should be conducted in larger cohorts with more bone oligometastatic patients to elucidate the relationship between TMB and these clinical characteristics or mutations.

The identification of new targetable drivers and the emergence of effective targeted therapies have greatly improved the clinical outcomes of patients with specific gene mutation. Here, we detected that 74.19% of bone oligometastatic patients had at least one actionable alteration according to OncoKB evidence. This percentage was slightly higher than the 67% of 1564 patients with usual advanced NSCLC reported in previous study [54], suggesting genetic alterations in bone oligometastasis are highly targetable. And the proportion of patients with level 1–2 actionable alterations as their highest actionable targets was also higher than the conventional advanced NSCLC patients (64.51% vs 57.1%) [54]. Matched targeted therapy could significantly prolong PFS and OS in NSCLC carrying level 1–2 genomic alterations, but no marked clinical benefit was observed in NSCLC with level 3–4 alterations [54]. The high prevalence of level 1–2 actionable alterations in bone oligometastasis may lead to better survival outcomes than in usual advanced NSCLC. Therefore, the use of more comprehensive genomic profiling to detect potentially actionable alterations is necessary in assisting treatment selection and improving the prognosis of patients with bone oligometastatic NSCLC.

We comprehensively depicted the genomic profiles of bone oligometastatic NSCLC and their correlation with TMB, as well as the distribution of actionable alterations. These findings may provide new insights to optimize personalized cancer treatment for guiding either targeted therapy or ICI treatment in bone oligometastatic NSCLC patients. Finally, our study has several limitations that are worth mentioning. First, this is a retrospective study with potential selection bias. Second, there is no accurate diagnostic standard of oligometastasis, so we may recruit polymetastatic tumors in our samples. Third, we calculated the TMB based on panel sequencing, which may not be as accurate as whole-exome sequencing. Performing whole-exome sequencing may better explore the relationship between genomic profile and TMB in future studies.

## Supplementary Information

**Additional file 1: Table S1.** Gene list of the 1021-gene panel.

**Additional file 2: Fig. S1.** **A** Mutational fraction of the six-base substitution for each sample. **B** Comparison of the high-frequency mutations identified in bone oligometastatic LUAD with that in the MSKCC cohort of LUAD. **C** Frequency distributions of EGFR. **D** Frequency of concurrent EGFR/TP53 mutations in bone oligometastasis. *p < 0.05, and **p < 0.01. **Fig. S2.** The mutated sites of genes with high mutation frequency, including EGFR (**A**), TP53 (**B**), KRAS (**C**), CDKN2A (**D**), MET (**E**), ARID2 (**F**), ATM (**G**), CTNNB1 (**H**), SMARCA4 (**I**).

## Data Availability

The raw sequence data generated and analyzed during the current study are available in the Genome Sequence Archive for Human repository, https://bigd.big.ac.cn/gsa-human/browse/HRA003878.

## Abbreviations

NSCLC: Non-small cell lung cancer
PFS: Progression-free survival
OS: Overall survival
TMB: Tumor mutation burden
NGS: Next-generation sequencing
FDA: Food and Drug Administration
FFPE: Formalin-fixed and paraffin-embedded
COSMIC: Catalogue of somatic mutations in cancer
SNVs: Single nucleotide variations
VAF: Variant allele fraction
GO: Gene ontology
KEGG: Kyoto encyclopedia of genes and genomes
ICI: Immune checkpoint inhibitor
TKIs: Tyrosine kinase inhibitors
LCT: Local consolidative therapy
HRD: Homologous recombination deficiency
LUAD: Lung adenocarcinoma
MSKCC: Memorial Sloan Kettering Cancer Center
