# Supplementary material for: Genomic features and its potential implication in bone oligometastatic NSCLC
Source: BMC Pulm Med. 2023 Feb 8;23:59. doi: 10.1186/s12890-023-02354-2 (PMC9906959; doi:10.1186/s12890-023-02354-2)
Supplement: Supplementary file 2 — Additional file 2: Fig. S1. A Mutational fraction of the six-base substitution for each sample. B Comparison of the high-frequency mutations identified in bone oligometastatic LUAD with that in the MSKCC cohort of LUAD. C Frequency distributions of EGFR. D Frequency of concurrent EGFR/TP53 mutations in bone oligometastasis. *p < 0.05, and **p < 0.01. Fig. S2. The mutated sites of genes with high mutation frequency, including EGFR (A), TP53 (B), KRAS (C), CDKN2A (D), MET (E), ARID2 (F), ATM (G), CTNNB1 (H), SMARCA4 (I). [file 12890_2023_2354_MOESM2_ESM.doc]

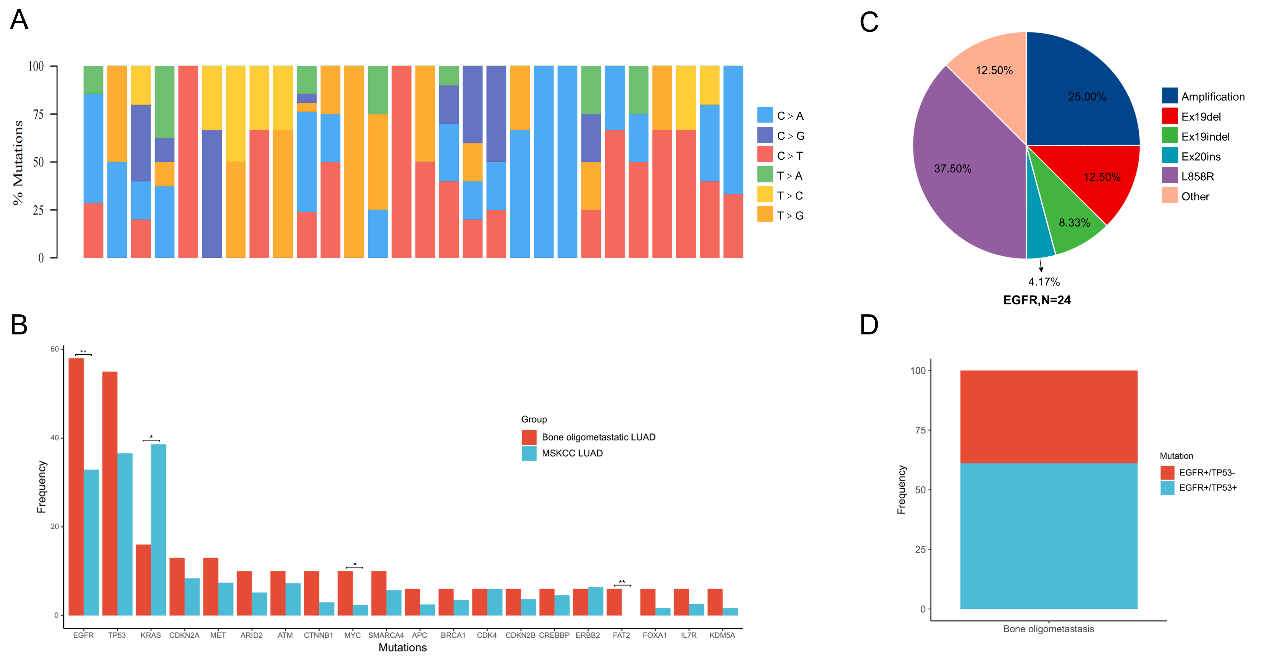


**Supplementary Figure 1. (A)** Mutational fraction of the six-base substitution for each sample. **(B)** Comparison of the high-frequency mutations identified in bone oligometastatic LUAD with that in the MSKCC cohort of LUAD. **(C)** Frequency distributions of EGFR. **(D)** Frequency of concurrent EGFR/TP53 mutations in bone oligometastasis. **p* < 0.05, and ***p* < 0.01.


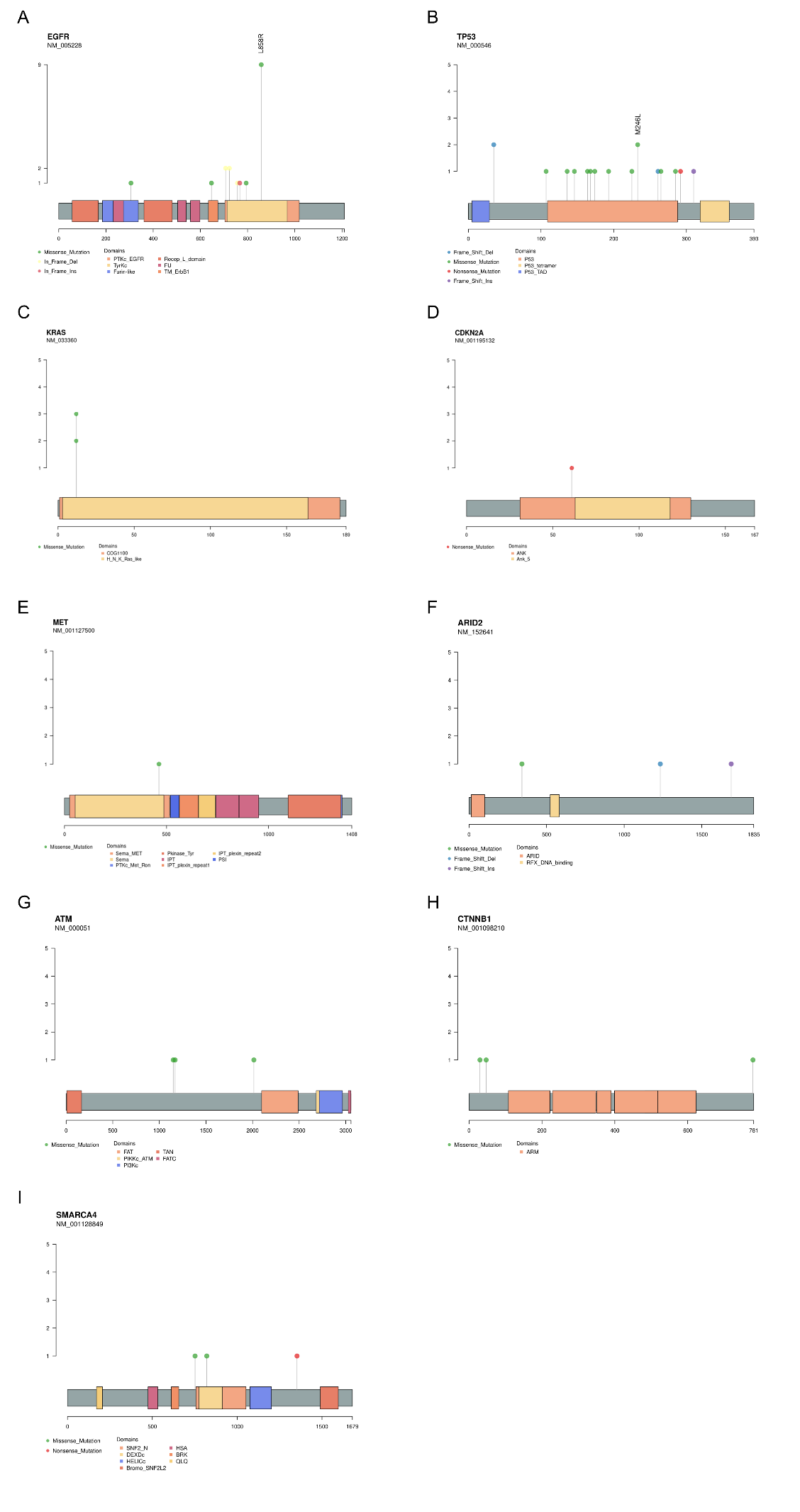


**Supplementary Figure 2.** The mutated sites of genes with high mutation frequency, including EGFR **(A)**, TP53 **(B)**, KRAS **(C)**, CDKN2A **(D)**, MET **(E)**, ARID2 **(F)**, ATM **(G)**, CTNNB1 **(H)**, SMARCA4 **(I)**.
